# Aging and inflammation limit the induction of SARS-CoV-2–specific CD8^+^ T cell responses in severe COVID-19

**DOI:** 10.1172/jci.insight.180867

**Published:** 2025-01-23

**Authors:** Gaëlle Autaa, Laura Papagno, Takuto Nogimori, Andrea Boizard-Moracchini, Daniil Korenkov, Maeva Roy, Koichiro Suzuki, Yuji Masuta, Eoghann White, Sian Llewellyn-Lacey, Yasuo Yoshioka, Francesco Nicoli, David A. Price, Julie Dechanet-Merville, Takuya Yamamoto, Isabelle Pellegrin, Victor Appay

**Affiliations:** 1 University of Bordeaux, CNRS UMR 5164, INSERM ERL 1303, ImmunoConcEpT, 33000 Bordeaux, France.; 2 Laboratory of Precision Immunology, Center for Intractable Diseases and ImmunoGenomics, National Institutes of Biomedical Innovation, Health and Nutrition, Osaka, Japan.; 3 CHU Bordeaux, Laboratory of Immunology and Immunogenetics, 33000 Bordeaux, France.; 4 The Research Foundation for Microbial Diseases of Osaka University (BIKEN), Osaka, Japan.; 5 Division of Infection and Immunity, Cardiff University School of Medicine, Cardiff, United Kingdom.; 6 Vaccine Creation Group, BIKEN Innovative Vaccine Research Alliance Laboratories, Research Institute for Microbial Diseases,; 7 Laboratory of Nano-Design for Innovative Drug Development, Graduate School of Pharmaceutical Sciences, and; 8 Institute for Open and Transdisciplinary Research Initiatives, Osaka University, Osaka, Japan.; 9 Department of Chemical, Pharmaceutical and Agricultural Sciences, University of Ferrara, Ferrara, Italy.; 10 Systems Immunity Research Institute, Cardiff University School of Medicine, Cardiff, United Kingdom.

**Keywords:** Aging, COVID-19, Immunology, Cellular immune response, T cells

## Abstract

CD8^+^ T cells are critical for immune protection against severe COVID-19 during acute infection with SARS-CoV-2. However, the induction of antiviral CD8^+^ T cell responses varies substantially among infected people, and a better understanding of the mechanisms that underlie such immune heterogeneity is required for pandemic preparedness and risk stratification. In this study, we analyzed SARS-CoV-2–specific CD4^+^ and CD8^+^ T cell responses in relation to age, clinical status, and inflammation among patients infected primarily during the initial wave of the pandemic in France or Japan. We found that age-related contraction of the naive lymphocyte pool and systemic inflammation were associated with suboptimal SARS-CoV-2–specific CD4^+^ and, even more evidently, CD8^+^ T cell immunity in patients with acute COVID-19. No such differences were observed for humoral immune responses targeting the spike protein of SARS-CoV-2. We also found that the proinflammatory cytokine IL-18, concentrations of which were significantly elevated among patients with severe disease, suppressed the de novo induction and memory recall of antigen-specific CD8^+^ T cells, including those directed against SARS-CoV-2. These results potentially explain the vulnerability of older adults to infections that elicit a profound inflammatory response, exemplified by acute COVID-19.

## Abstract 2

The study provides new insights into the development of severe COVID-19 and highlights potential therapeutic options that could favorably influence disease outcome, especially in older people, who are particularly vulnerable to infections with emerging pathogens.

## Introduction

Infection with ancestral strains of SARS-CoV-2 resulted in severe disease in up to 5% of cases, accompanied by a high rate of mortality (1). Advanced age subsequently emerged as a prominent risk factor for disease severity and death (2). Our understanding of the mechanisms underlying this association has nonetheless remained incomplete, limiting the development of vaccines that effectively protect older people from acute COVID-19.

Systemic inflammation is a hallmark of severe COVID-19 (3, 4). Moreover, the acute cytokine storm characteristic of life-threatening disease often occurs in the context of high viral loads and delayed viral clearance (5, 6), implying suboptimal immune control of SARS-CoV-2. Although neutralizing antibodies play a key role in preventing infection, T cells are indispensable for limiting viral replication and disease progression (7), especially soon after the acquisition of SARS-CoV-2 (8–15). Several lines of evidence indicate that CD8^+^ T cells in particular are required for immune protection against severe COVID-19. For example, the emergence of viral variants that escape CD8^+^ T cell recognition suggests immune pressure targeting epitopes restricted by HLA class I molecules (16–18), a specific allotype of which has been linked with asymptomatic cases of infection with SARS-CoV-2 (19). Antibody-mediated depletion experiments in nonhuman primates have also shown more directly that CD8^+^ T cells contribute to immune control of SARS-CoV-2 (20). In addition, patients with mild disease typically mount highly cytotoxic, polyfunctional SARS-CoV-2–specific CD8^+^ T cell responses, linking immune effector potency with the outcome of acute COVID-19 (21–24).

SARS-CoV-2–specific CD4^+^ and CD8^+^ T cell responses vary among individuals during acute infection (9, 25) and remain undetectable in 30% of patients with acute COVID-19 (26). We explored the mechanistic basis of this immune heterogeneity in separate cohorts of unvaccinated patients infected mainly during the first wave of the pandemic in France or Japan. Our data revealed that age-related contraction of the naive lymphocyte pool and systemic inflammation undermined the induction of effective SARS-CoV-2–specific CD4^+^ and, more profoundly, CD8^+^ T cell immunity in older people with acute COVID-19, highlighting a potentially key role for the proinflammatory cytokine IL-18.

## Results

### SARS-CoV-2–specific CD8^+^ T cells are quantitatively and qualitatively suboptimal in patients with severe COVID-19.

To identify the factors that shape adaptive immunity across the spectrum of disease in patients with acute COVID-19, we evaluated virus-specific antibody responses and CD4^+^ and CD8^+^ T cell responses in unvaccinated donors infected predominantly during the early phase of the pandemic with ancestral strains of SARS-CoV-2. Data were correlated with age, clinical status, and markers of inflammation spanning a primary cohort of individuals recruited from France and a secondary cohort of individuals recruited from Japan (Table 1). This approach was designed to account for geographical and technical variables that could potentially confound data interpretation. Importantly, dexamethasone was not included as standard care at the time, allowing investigation in the absence of iatrogenic immunosuppression.

**Figure 1:**
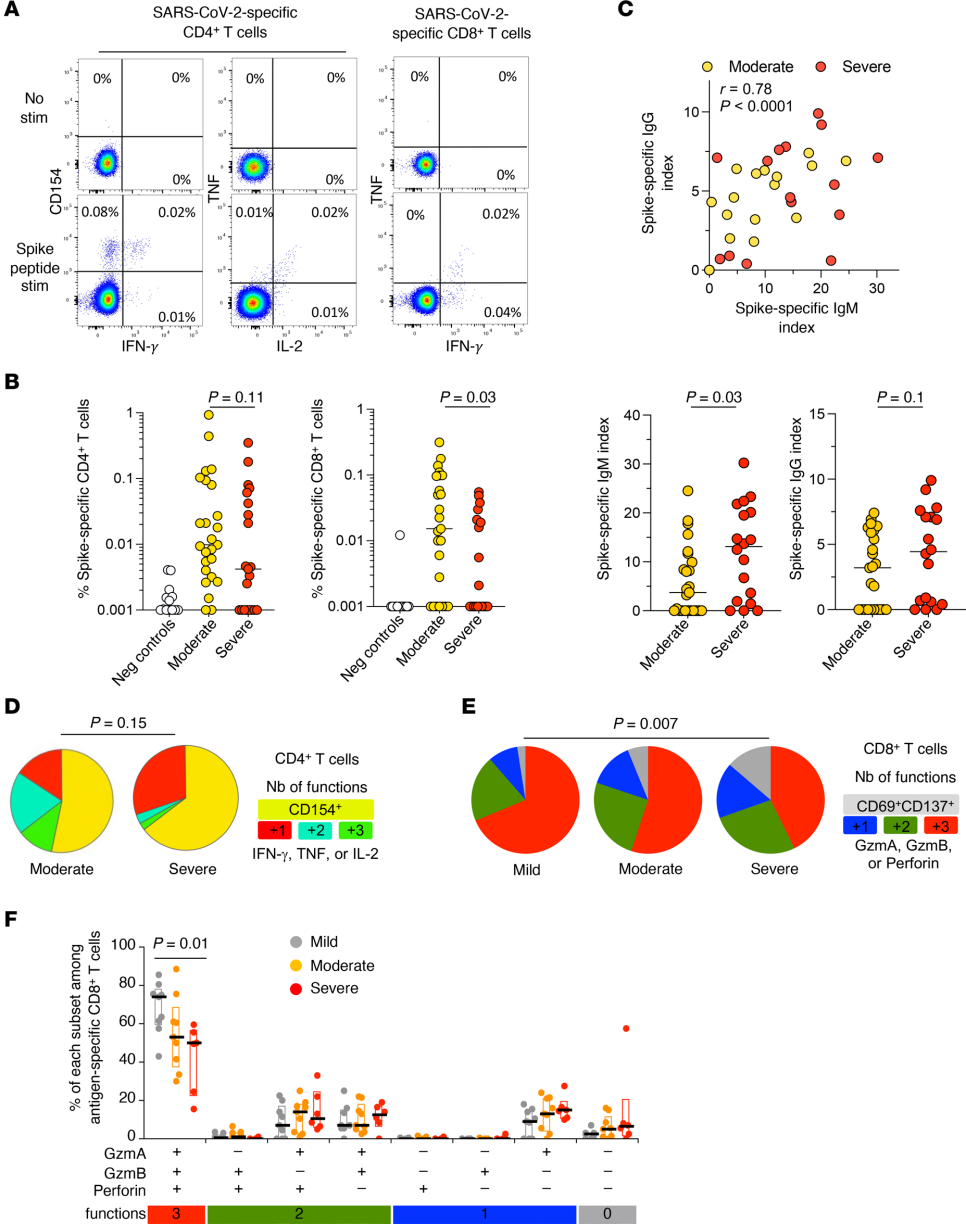
Characterization of SARS-CoV-2–specific CD4^+^ and CD8^+^ T cells and humoral immunity in patients with acute COVID-19. (**A**) Representative flow cytometry plots showing the identification of SARS-CoV-2–specific CD4^+^ and CD8^+^ T cells after stimulation of PBMCs with overlapping spike peptides on day 4 after hospitalization. Plots are gated on CD4^+^ (left and center) or CD8^+^ T cells (right). (**B**) Frequencies of spike-specific CD4^+^ (CD154^+^) or CD8^+^ T cells (IFN-γ^+^) among uninfected controls and patients in the primary cohort grouped according to disease severity. Each dot represents 1 donor. Bars indicate median values. Significance was assessed using the Mann-Whitney *U* test. (**C**) Top: Correlation between SARS-CoV-2–specific IgM and IgG titers among patients in the primary cohort with moderate or severe disease. Each dot represents 1 donor. Significance was assessed using Spearman’s rank test. Bottom: SARS-CoV-2–specific IgM (left) and IgG titers (right) among patients in the primary cohort grouped according to disease severity. Each dot represents 1 donor. Bars indicate median values. Significance was assessed using the Mann-Whitney *U* test. (**D**) Functional profiles of spike-specific CD4^+^ T cells among patients in the primary cohort grouped according to disease severity (moderate disease, *n* = 23; severe disease, *n* = 14). Pie chart segments indicate the numbers of expressed functions color-matched to the key. Significance was assessed using a permutation test. (**E**) Functional profiles of spike-specific CD8^+^ T cells among patients in the secondary cohort grouped according to disease severity (mild disease, *n* = 9; moderate disease, *n* = 9; severe disease, *n* = 6). Pie chart segments indicate the numbers of expressed functions color-matched to the key. Significance was assessed using a permutation test. (**F**) Combinatorial analysis of spike-specific CD8^+^ T cell functionality among patients in the secondary cohort grouped according to disease severity. Each dot represents 1 donor. Bars indicate median values, and boxes indicate upper and lower quartile values. Significance was assessed using the Mann-Whitney *U* test.

In the primary cohort, PBMCs were stimulated with overlapping peptides representing the full length of the viral spike protein, and SARS-CoV-2–specific CD4^+^ and CD8^+^ T cells were identified functionally via upregulation of the activation marker CD154 and/or intracellular production of the effector cytokines IFN-γ, TNF, or IL-2 (Figure 1A and Supplemental Figure 1, A and B; supplemental material available online with this article; https://doi.org/10.1172/jci.insight.180867DS1). SARS-CoV-2–specific CD8^+^ T cell frequencies were higher in patients with moderate versus severe COVID-19 (Figure 1B). A similar trend was observed for SARS-CoV-2–specific CD4^+^ T cells without achieving significance (Figure 1B). This pattern was replicated in the secondary cohort using a similar approach to enumerate SARS-CoV-2–specific CD4^+^ and CD8^+^ T cells functionally via upregulation of the activation markers CD69 and CD137 (Supplemental Figure 1C), with concomitant intracellular measurements of the cytotoxins granzyme A, granzyme B, and perforin (Supplemental Figure 1D). These data suggested that severe disease was associated with quantitatively impaired SARS-CoV-2–specific CD8^+^ T cell immunity (Supplemental Figure 2A). However, it should be noted that we were unable to assess the possibility that SARS-CoV-2–specific CD8^+^ T cells preferentially trafficked to the site of infection in patients with severe COVID-19, a phenomenon that could provide some degree of localized immune protection (27). No associations with disease severity were detected for spike-specific IgM or IgG titers or neutralizing antibody activity against SARS-CoV-2 (Figure 1C and Supplemental Figure 2B).

The intracellular detection of cytokines and cytotoxins further enabled us to evaluate the effector profiles of SARS-CoV-2–specific CD4^+^ and CD8^+^ T cells in the primary and secondary cohorts, respectively. In the primary cohort, SARS-CoV-2–specific CD4^+^ T cells were more polyfunctional in patients with moderate versus severe disease, albeit without achieving significance (Figure 1D), and in the secondary cohort, SARS-CoV-2–specific CD8^+^ T cells were more polyfunctional in patients with mild versus severe disease (Figure 1E). Moreover, the lowest frequencies of SARS-CoV-2–specific CD8^+^ T cells expressing granzyme A, granzyme B, and perforin in combination were detected in patients with severe disease (Figure 1F). These data suggested that severe disease was associated with qualitatively impaired SARS-CoV-2–specific CD8^+^ T cell immunity.

### Age-related contraction of the naive lymphocyte pool limits the induction of SARS-CoV-2–specific CD8^+^ T cells in patients with acute COVID-19.

**Figure 2:**
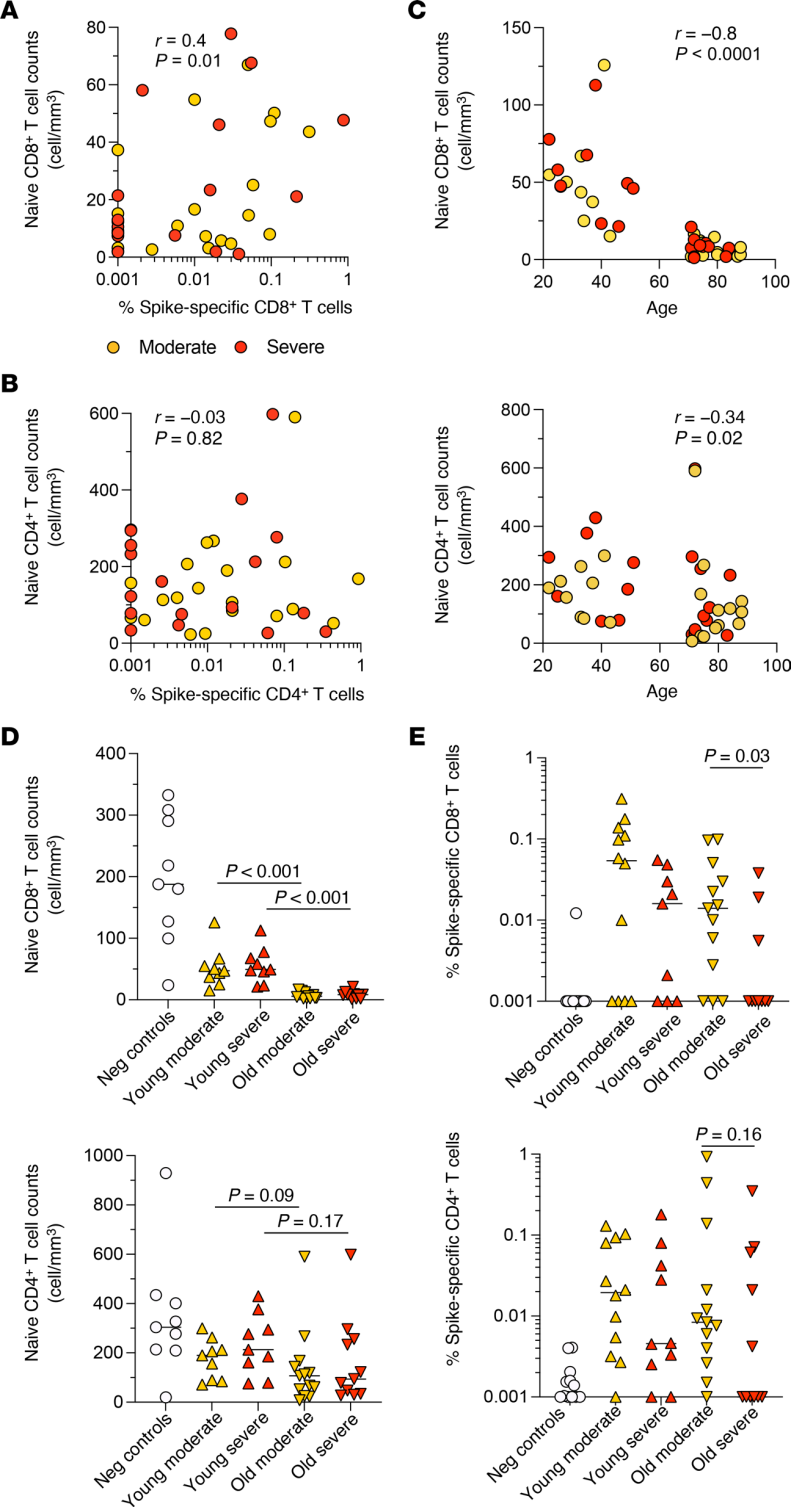
Impact of age and disease severity on SARS-CoV-2–specific CD4^+^ and CD8^+^ T cells in patients with acute COVID-19. (**A**) Correlation between the absolute counts of naive CD8^+^ T cells and the frequencies of spike-specific CD8^+^ T cells among patients in the primary cohort with moderate or severe disease. Each dot represents 1 donor. Significance was assessed using Spearman’s rank test. (**B**) Correlation between the absolute counts of naive CD4^+^ T cells and the frequencies of spike-specific CD4^+^ T cells among patients in the primary cohort with moderate or severe disease. Each dot represents 1 donor. Significance was assessed using Spearman’s rank test. (**C**) Correlations between the absolute counts of naive CD8^+^ (top) or CD4^+^ T cells (bottom) and age among patients in the primary cohort with moderate or severe disease. Each dot represents 1 donor. Significance was assessed using Spearman’s rank test. (**D**) Absolute counts of naive CD8^+^ (top) or CD4^+^ T cells (bottom) among patients in the primary cohort grouped according to age and disease severity. Each dot represents 1 donor. Bars indicate median values. Significance was assessed using the Mann-Whitney *U* test. (**E**) Frequencies of spike-specific CD8^+^ (top) or CD4^+^ T cells (bottom) among patients in the primary cohort grouped according to age and disease severity. Each dot represents 1 donor. Bars indicate median values. Significance was assessed using the Mann-Whitney *U* test.

In further analyses, we detected an inverse correlation between SARS-CoV-2–specific CD8^+^ T cell frequencies and age (Supplemental Figure 3A). No such correlations were observed for SARS-CoV-2–specific CD4^+^ T cell frequencies (Supplemental Figure 3A) or spike-specific antibody titers (IgM or IgG) (Supplemental Figure 3B). The induction of CD8^+^ T cell responses is contingent on the presence of naive antigen-specific precursors in the periphery, the quantity and quality of which can vary with age. Sample limitations dictated that we were unable to measure the frequencies of naive SARS-CoV-2–specific CD8^+^ T cells reproducibly. The absolute counts of naive CD8^+^ T cells nonetheless correlated directly with the frequencies of SARS-CoV-2–specific CD8^+^ T cells in both cohorts of patients with acute COVID-19 (Figure 2A and Supplemental Figure 2C). No such correlations were detected among CD4^+^ T cells in patients with acute COVID-19, irrespective of the recruitment site (Figure 2B and Supplemental Figure 2D). It should be noted that we did not formally exclude stem cell–like memory cells from our naive cell gate based on the expression of CD95. However, the frequencies of stem cell–like memory CD8^+^ T cells are generally very low (28) and typically increase with age (29), whereas the frequencies of naive CD8^+^ T cells almost invariably decline with age (30). This latter phenomenon was confirmed here (Figure 2C and Supplemental Figure 2E).

In addition to a numerical decline with age, naive CD8^+^ T cells exhibit functional impairments in older people, notably including a reduced capacity to differentiate and proliferate in response to antigen (31, 32). We found that naive CD8^+^ T cells from older people, including patients with acute COVID-19, proliferated to a lesser extent in response to stimulation and exhibited increased levels of β-galactosidase activity compared with naive CD8^+^ T cells from younger people, again including patients with acute COVID-19 (Supplemental Figure 4, A and B). Senescence-associated β-galactosidase activity also correlated inversely with the absolute counts of naive CD8^+^ T cells and the frequencies of SARS-CoV-2–specific CD8^+^ T cells in patients with acute COVID-19 (Supplemental Figure 4C).

These collective data suggested that quantitative and qualitative deficiencies in the naive CD8^+^ T cell pool limited the induction of SARS-CoV-2–specific CD8^+^ T cells in patients with acute COVID-19. However, the absolute counts of naive CD8^+^ T cells were relatively low in older people, irrespective of disease severity (Figure 2D), and the frequencies of SARS-CoV-2–specific CD8^+^ T cells were higher in older people with moderate disease versus older people with severe disease (Figure 2E). Similar patterns were observed for SARS-CoV-2–specific CD4^+^ T cells without achieving significance (Figure 2E). Accordingly, there was no strict relationship between immune aging and disease severity, suggesting that other factors could impact the relationship between SARS-CoV-2–specific CD8^+^ T cell immunity and the outcome of acute COVID-19.

### Disease-associated inflammation limits the induction of SARS-CoV-2–specific CD8^+^ T cells in patients with severe COVID-19.

**Figure 3:**
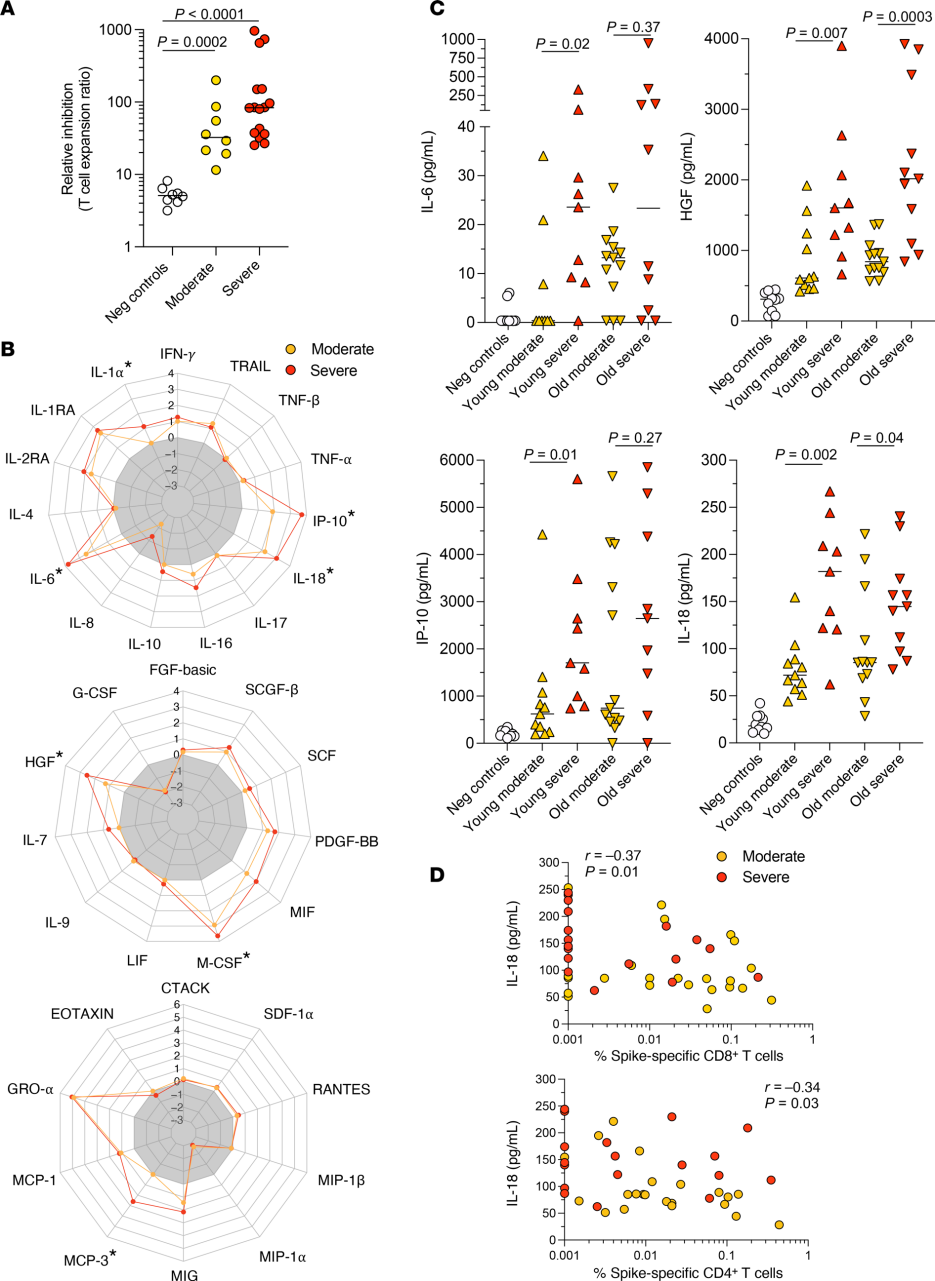
Cytokine profiles in patients with acute COVID-19. (**A**) Effect of serum from uninfected controls or patients in the primary cohort with moderate or severe disease on the expansion of GIL-specific CD8^+^ T cells in vitro. Relative inhibition was calculated as the ratio of GIL/HLA-A*02:01 tetramer^+^ CD8^+^ T cell frequencies on day 12 after peptide stimulation of PBMCs from healthy HLA-A2^+^ donors in the absence/presence of serum. Each dot represents 1 experiment. Bars indicate median values. Significance was assessed using Wilcoxon’s signed-rank test. (**B**) Radar plots showing the mean plasma concentrations of proinflammatory cytokines (top), homeostatic cytokines (middle), and various chemokines (bottom) among patients in the primary cohort with moderate (*n* = 25) or severe disease (*n* = 20). Results are expressed relative to the corresponding values among uninfected controls (*n* = 10). ^*^P < 0.01 by Mann-Whitney *U* test. (**C**) Plasma concentrations of IL-6, HGF, IP-10, and IL-18 among patients in the primary cohort grouped according to age and disease severity. Each dot represents 1 donor. Bars indicate median values. Significance was assessed using the Mann-Whitney *U* test. (**D**) Correlations between the frequencies of spike-specific CD8^+^ (top) or CD4^+^ T cells (bottom) and plasma concentrations of IL-18 among patients in the primary cohort with moderate or severe disease. Each dot represents 1 donor. Significance was assessed using Spearman’s rank test.

To determine whether the inflammatory environment associated with acute disease could impair the induction of SARS-CoV-2–specific CD8^+^ T cells, we stimulated PBMCs from healthy HLA-A2^+^ donors in vitro with the influenza virus M1 peptide GILGFVFTL (GIL, residues 58–66) in the presence of serum from uninfected controls or patients with moderate or severe COVID-19. The expansion of M1-specific CD8^+^ T cells after 12 days in culture was quantified using tetrameric complexes of GIL/HLA-A*02:01. In this experimental setting, the expansion of CD8^+^ T cells in response to antigen-mediated stimulation was inhibited in the presence of serum from patients with acute COVID-19, especially those with severe disease (Figure 3A).

We then measured the concentrations of some chemokines, growth factors, and proinflammatory cytokines in plasma samples from patients in the primary cohort to gain insights into the soluble factor(s) that might be responsible for this inhibitory effect on antigen-specific CD8^+^ T cell expansion. As expected, plasma concentrations of several inflammatory mediators, including IL-1α, IL-6, IL-18, IP-10, HGF, MCP-3, and M-CSF, were elevated in patients with acute COVID-19, especially those with severe disease (Figure 3B). Moreover, plasma concentrations of IL-6 and IP-10 and, even more strikingly, IL-18 and HGF were higher in patients with severe versus moderate disease, irrespective of age (Figure 3C). No correlations were detected between the plasma concentrations of any of these inflammatory mediators and age (Supplemental Figure 3C). Similar trends were observed in the secondary cohort, although we acquired too few data points to achieve significance (Supplemental Figure 2F). We also measured plasma concentrations of IL-18 binding protein (IL-18BP), a decoy receptor for IL-18 (33). These assays enabled us to calculate the concentrations of free IL-18, which were similarly elevated as a function of disease severity (Supplemental Figure 5A). Importantly, the frequencies of SARS-CoV-2–specific CD8^+^ T cells correlated inversely with plasma concentrations of IL-18 and HGF (Figure 3D and Supplemental Figure 6A), and the frequencies of SARS-CoV-2–specific CD4^+^ T cells correlated inversely with plasma concentrations of IL-6, IL-18, IP-10, and HGF (Figure 3D and Supplemental Figure 6B). These observations suggested that certain inflammatory mediators could inhibit the induction of SARS-CoV-2–specific CD4^+^ and CD8^+^ T cells in patients with acute COVID-19.

**Figure 4:**
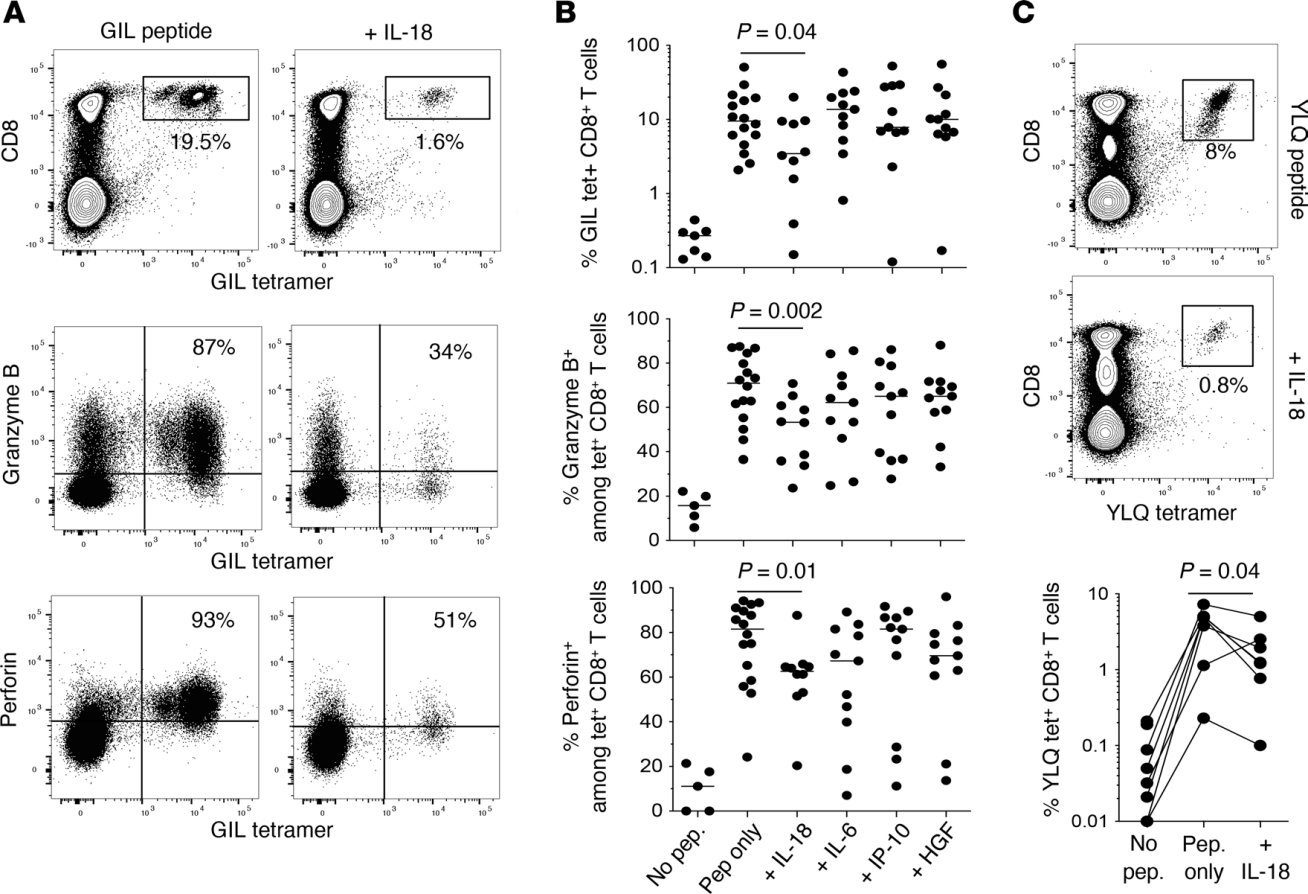
Impact of IL-18 on recall CD8^+^ T cell responses in vitro. (**A**) Top: Representative flow cytometry plots showing the identification of GIL/HLA-A*02:01 tetramer^+^ CD8^+^ T cells on day 12 after peptide stimulation of PBMCs from healthy HLA-A2^+^ donors in the absence (left) or presence of IL-18 (right). Plots are gated on viable lymphocytes. Middle and bottom: Intracellular expression of granzyme B (middle) or perforin (bottom) among GIL/HLA-A*02:01 tetramer^+^ CD8^+^ T cells expanded in the absence (left) or presence of IL-18 (right). Plots are gated on CD8^+^ T cells. (**B**) Top: Frequencies of GIL-specific CD8^+^ T cells expanded in the absence or presence of IL-18, IL-6, IP-10, or HGF. Middle and bottom: Intracellular expression of granzyme B (middle) or perforin (bottom) among GIL/HLA-A*02:01 tetramer^+^ CD8^+^ T cells expanded in the absence or presence of IL-18, IL-6, IP-10, or HGF. Details as in **A**. Unstimulated controls are shown for reference. Each dot represents 1 donor. Bars indicate median values. Significance was assessed using Wilcoxon’s signed-rank test. (**C**) Top and middle: Representative flow cytometry plots showing the identification of YLQ/HLA-A*02:01 tetramer^+^ CD8^+^ T cells on day 12 after peptide stimulation of PBMCs from healthy HLA-A2^+^ donors in the absence (top) or presence of IL-18 (middle). Plots are gated on viable lymphocytes. Bottom: Frequencies of YLQ-specific CD8^+^ T cells expanded in the absence or presence of IL-18. Unstimulated controls are shown for reference. Each dot represents 1 donor. Significance was assessed using Wilcoxon’s signed-rank test.

To test this hypothesis, we used an approach based on an accelerated dendritic cell coculture system to study the activation and proliferation of antigen-specific CD8^+^ T cells in vitro in the presence of IL-6, IL-18, IP-10, or HGF (34). The expansion of memory GIL-specific CD8^+^ T cells in response to stimulation with the cognate peptide was reduced in the presence of IL-18 but not in the presence of IL-6, IP-10, or HGF (Figure 4, A and B). GIL-specific CD8^+^ T cells expanded in the absence of IL-18 also expressed the cytotoxins granzyme B and perforin more commonly than GIL-specific CD8^+^ T cells expanded in the presence of IL-18 (Figure 4, A and B). No such differences were observed for GIL-specific CD8^+^ T cells expanded in the presence of IL-6, IP-10, or HGF (Figure 4, A and B). Similarly, the expansion of memory CD8^+^ T cells specific for the immunodominant HLA-A2–restricted SARS-CoV-2 spike epitope YLQPRTFLL (YLQ, residues 269–277) (17, 35) in response to stimulation with the cognate peptide was reduced in the presence of IL-18, extending our findings to a different specificity (Figure 4C).

**Figure 5:**
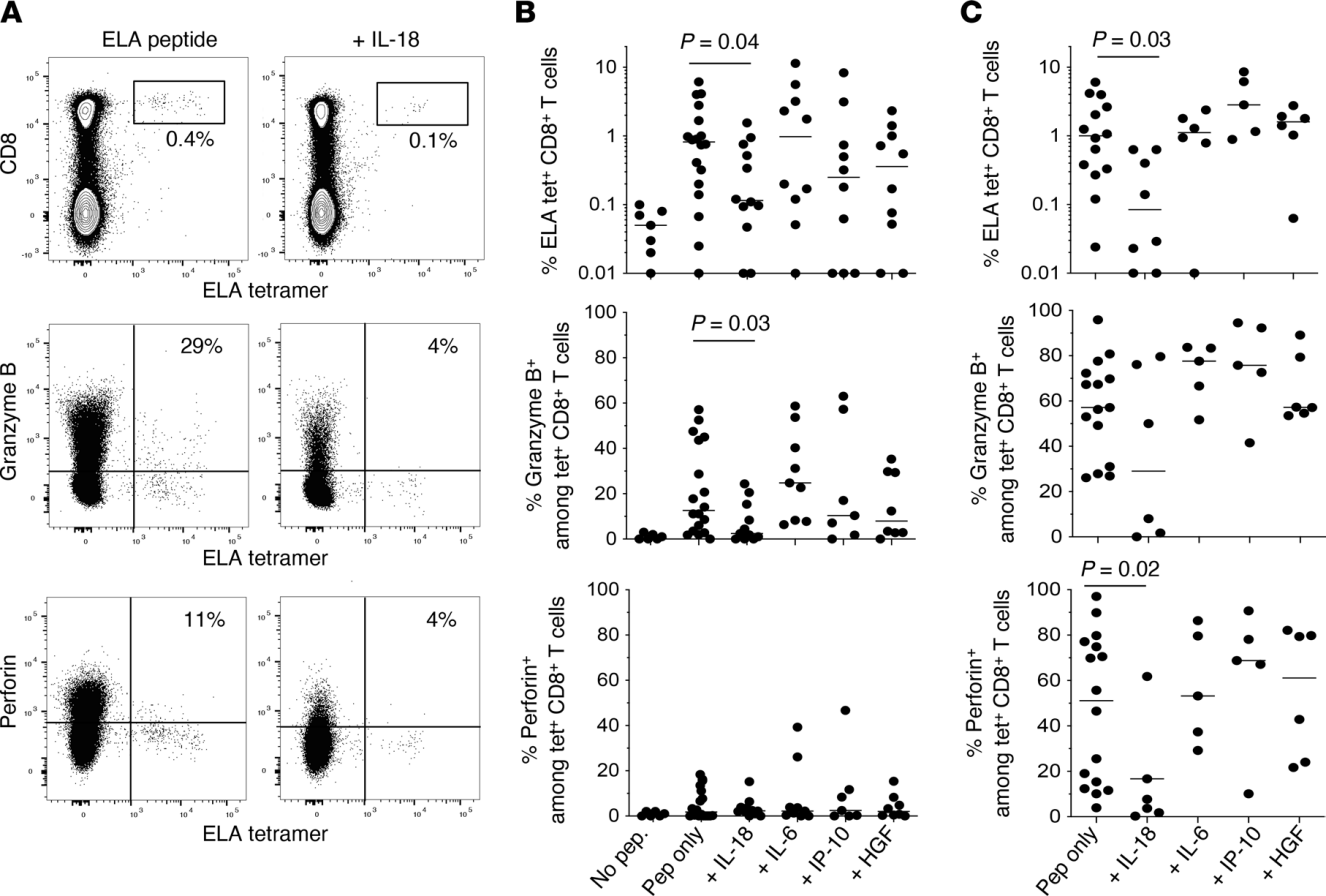
Impact of IL-18 on de novo CD8^+^ T cell responses in vitro. (**A**) Top: Representative flow cytometry plots showing the identification of ELA/HLA-A*02:01 tetramer^+^ CD8^+^ T cells on day 12 after peptide stimulation of PBMCs from healthy HLA-A2^+^ donors in the absence (left) or presence of IL-18 (right). Plots are gated on viable lymphocytes. Middle and bottom: Intracellular expression of granzyme B (middle) or perforin (bottom) among ELA/HLA-A*02:01 tetramer^+^ CD8^+^ T cells expanded in the absence (left) or presence of IL-18 (right). Plots are gated on CD8^+^ T cells. (**B**) Top: Frequencies of ELA-specific CD8^+^ T cells expanded in the absence or presence of IL-18, IL-6, IP-10, or HGF. Middle and bottom: Intracellular expression of granzyme B (middle) or perforin (bottom) among ELA/HLA-A*02:01 tetramer^+^ CD8^+^ T cells expanded in the absence or presence of IL-18, IL-6, IP-10, or HGF. Details as in **A**. Unstimulated controls are shown for reference. Each dot represents 1 donor. Bars indicate median values. Significance was assessed using Wilcoxon’s signed-rank test. (**C**) Replicate experiments performed in the presence of single-stranded RNA. Other details as in **B**.

In further experiments, we assessed the influence of IL-18 on the de novo induction of CD8^+^ T cells, which is potentially more relevant than recall responses in the context of primary infection with SARS-CoV-2. For this purpose, we focused on a model antigen, namely the heteroclitic HLA-A2–restricted tumor-associated epitope ELAGIGILTV (ELA, Melan-A/MART-1_26–35_). Large numbers of naive precursors recognize this antigen in individuals expressing HLA-A2 (31, 36), facilitating experimental reproducibility. The expansion of the ELA-specific CD8^+^ T cells in response to stimulation with the cognate peptide was reduced in the presence of IL-18 but not in the presence of IL-6, IP-10, or HGF (Figure 5, A and B). ELA-specific CD8^+^ T cells expanded in the absence of IL-18 also expressed granzyme B more commonly than ELA-specific CD8^+^ T cells expanded in the presence of IL-18. Similar patterns of expansion were observed in otherwise identical assays supplemented with single-stranded RNA, a ligand for TLR8, which was included to mimic innate stimuli provided by SARS-CoV-2 (Figure 5C). ELA-specific CD8^+^ T cells expanded in the absence of IL-18 also expressed perforin more commonly under these conditions than ELA-specific CD8^+^ T cells expanded in the presence of IL-18 (Figure 5C). Importantly, the addition of IL-18BP and an anti–IL-18 receptor antibody restored the expansion of activated CD8^+^ T cells specific for GIL, YLQ, or ELA (Supplemental Figure 5B).

These collective data suggested that proinflammatory mediators characteristic of severe disease, most notably IL-18, limited the induction and recall of highly cytotoxic SARS-CoV-2–specific CD8^+^ T cells in patients with acute COVID-19.

## Discussion

In this study, we evaluated the induction of SARS-CoV-2–specific CD4^+^ and, more pertinently, CD8^+^ T cells as a function of age and disease severity in unvaccinated patients with acute COVID-19 recruited across separate cohorts from France and Japan. CD8^+^ T cells in particular are known to mediate immune protection against intracellular pathogens (37), a scenario that likely extends to SARS-CoV-2. Importantly, our approach enabled us to validate biological associations across geographical boundaries and technical variables, minimizing investigational uncertainty. In addition, we controlled indirectly for viral sequence variation, given that all patients were infected early during the pandemic with ancestral strains of SARS-CoV-2.

Our first key finding was that age-related contraction of the naive lymphocyte pool and, by extension, the frequencies of antigen-specific precursors limited the induction of SARS-CoV-2–specific CD8^+^ T cells across the spectrum of disease severity in patients with acute COVID-19. Similar data have been reported previously, both in vitro (31, 35, 38) and ex vivo in the context of vaccination against SARS-CoV-2 (39, 40). Qualitative deficits in the naive lymphocyte pool were also associated with impaired SARS-CoV-2–specific CD8^+^ T cell responses in older people. In contrast, the induction of SARS-CoV-2–specific CD4^+^ T cells was impacted to a lesser extent by age, potentially reflecting better preservation of the corresponding naive lymphocyte pool and/or greater recognition degeneracy.

Our second key finding was that disease-associated inflammation, a distinctive feature of primary infection (41), inhibited the induction of SARS-CoV-2–specific CD4^+^ and CD8^+^ T cells in patients with acute COVID-19. In particular, we found that IL-18, which circulates at high concentrations in patients with severe disease, impaired the expansion and functionality of antigen-specific CD8^+^ T cells during induction and recall from memory. It should be noted that our findings were derived from experiments performed in vitro, which likely did not capture the full complexity of the inflammatory environment in vivo, and associations detected ex vivo, which highlighted similar inverse correlations for other soluble factors, including HGF. Moreover, inflammatory mediators beyond those measured here could have contributed to the observed suppression of adaptive CD8^+^ T cell immunity, potentially acting in concert with IL-18. The mechanisms underlying these effects require further elucidation. It should also be noted that we did not determine the cellular source(s) of IL-18 in patients with acute COVID-19.

IL-18 has emerged as a strong predictive marker of disease severity in patients with acute COVID-19 (33, 42–44). Similar findings have been reported previously for other acute viral diseases, such as influenza and dengue (45, 46). Accordingly, interventions targeting the activity of IL-18 may enhance viral clearance in these contexts by releasing constraints on the induction of adaptive CD8^+^ T cell immunity, as suggested by the findings reported here. One potential drug candidate for this purpose is recombinant human IL-18BP, which has already found clinical utility in other diseases associated with elevated systemic concentrations of IL-18 (47, 48). Conversely, autoimmune diseases associated with aberrant CD8^+^ T cell immunity, such as vitiligo, may benefit from interventions designed to enhance the activity of IL-18 (49).

In summary, we have shown that advanced age and systemic inflammation can suppress SARS-CoV-2–specific CD4^+^ and, to a greater extent, CD8^+^ T cell immunity in patients with acute COVID-19. We also identified a key inhibitory role in this process for the proinflammatory cytokine IL-18. Our findings potentially explain why older people with acute inflammatory syndrome are prone to severe disease and death after infection with SARS-CoV-2. It is intriguing to speculate that therapies directed against IL-18 may enhance SARS-CoV-2–specific CD4^+^ and CD8^+^ T cell immunity, not only during acute infection but also during convalescence to help clear viral reservoirs in patients with long COVID.

## Methods

### Sex as a biological variable.

Male and female patients were eligible for inclusion. Biological parameters were evaluated collectively. Patient characteristics are summarized in Table 1.

### Patients and samples.

Peripheral blood samples were obtained from unvaccinated adults infected with ancestral strains of SARS-CoV-2 at the beginning of the pandemic in France or Japan. Patients were treated with supportive care only, eliminating the potential confounding influence of iatrogenic immunosuppression. Clinical and demographic data were recorded for all patients, including age, sex, admission diagnosis, medications, and the incidence of comorbidities, such as hypertension, diabetes, congestive cardiac failure, chronic kidney disease, and chronic lung disease. Patients were categorized into 3 subgroups according to diagnostic guides from the French and Japanese Ministries of Health: (a) mild (no respiratory symptoms, SpO_2_ ≥ 96%); (b) moderate (with or without dyspnea and without oxygen support, 93% < SpO_2_ < 96%); or (c) severe (with oxygen support, SpO_2_ ≤ 93%). Healthy uninfected donors were recruited among health care staff at each site. PBMCs were isolated using standard density gradient centrifugation with Ficoll-Paque PLUS (Cytiva) or BD Vacutainer CPT Cell Preparation Tubes with EDTA (BD Biosciences) and then cryopreserved at –150°C.

### Standard immune monitoring.

T cells were phenotyped ex vivo via flow cytometry using directly conjugated antibodies specific for CCR7 (clone 3D12, BD Biosciences), CD4 (clone L200, BD Biosciences), CD8 (clone SK1, BD Biosciences), CD27 (clone O323, BioLegend), and CD45RA (clone HI100, eBioscience). Nonviable events were excluded using a LIVE/DEAD Fixable Aqua Dead Cell Stain Kit (Thermo Fisher Scientific). Data were acquired using an LSR Fortessa (BD Biosciences) and analyzed using FlowJo version 10.8.1 (FlowJo LLC). Spike-specific IgM and IgG titers in serum samples from patients in the primary cohort were measured using an ARCHITECT i2000SR Immunoassay (Abbott). In-house assays were used to measure spike-specific IgG titers and SARS-CoV-2 neutralization activity in serum samples from patients recruited in Japan. Inflammatory mediators were quantified in plasma samples from patients in the primary or secondary cohorts using a Bio-Plex Pro Human Cytokine Screening Panel (Bio-Rad) or a ProcartaPlex Immunoassay (Thermo Fisher Scientific) in conjunction with a Luminex 200 System (Thermo Fisher Scientific), respectively.

### Ex vivo characterization of SARS-CoV-2–specific CD4^+^ and CD8^+^ T cells.

PBMCs were thawed and rested for 2 hours in RPMI 1640 medium containing 10% fetal bovine serum (FBS) and 1% penicillin/streptomycin (all reagents from Thermo Fisher Scientific). Cells were then incubated in the absence or presence of overlapping peptides spanning the full length of the SARS-CoV-2 spike protein, each at a final concentration of 2 μg/mL (Miltenyi Biotec). After 1 hour, protein transport was blocked using GolgiPlug (1 μL/mL, BD Biosciences) and GolgiStop (0.7 μL/mL, BD Biosciences), and cells were cultured for a further 6 hours to allow intracellular cytokine/cytotoxin accumulation. Cells from patients in the primary cohort were washed and stained with directly conjugated antibodies specific for CCR7 (clone G043H7, BioLegend), CD3 (clone REA613, Miltenyi Biotec), CD4 (clone REA623, Miltenyi Biotec), CD8 (clone REA734, Miltenyi Biotec), and CD45RA (clone REA1047, Miltenyi Biotec). Nonviable events were excluded using a LIVE/DEAD Fixable Aqua Dead Cell Stain Kit (Thermo Fisher Scientific). Cells were then fixed/permeabilized using an Inside Stain Kit (Miltenyi Biotec) and stained with directly conjugated antibodies specific for CD154 (clone REA238, Miltenyi Biotec), IFN-γ (clone REA600, Miltenyi Biotec), TNF (clone MAb11, BioLegend), and IL-2 (clone REA689, Miltenyi Biotec). Cells from patients in the secondary cohort were washed and stained with directly conjugated monoclonal antibodies specific for CD3 (clone SP34-2, BD Biosciences), CD4 (clone L200, BD Biosciences), CD8 (clone RPA-T8, BD Biosciences), CD27 (clone 1A4CD27, Beckman Coulter), and CD45RO (clone UCHL1, BD Biosciences). Nonviable events were excluded using a LIVE/DEAD Fixable Blue Dead Cell Stain Kit (Thermo Fisher Scientific). Cells were then fixed/permeabilized using a Cytofix/Cytoperm Kit (BD Biosciences) and stained with directly conjugated monoclonal antibodies specific for CD69 (clone FN50, BD Biosciences), CD137 (clone 4B4-1, BioLegend), granzyme A (clone CB9, BioLegend), granzyme B (clone GB11, Thermo Fisher Scientific), and perforin (clone B-D48, BioLegend). Data were acquired using an LSR Fortessa (BD Biosciences) or a FACSymphony A5 (BD Biosciences) and analyzed using FlowJo version 10.8.1 (FlowJo LLC).

### Quantification of free IL‑18.

Plasma concentrations of IL-18 were quantified using a Human Total IL-18/IL-1F4 Quantikine ELISA (R&D Systems), and plasma concentrations of IL-18BP were quantified using a Human IL-18 BPa Quantikine ELISA (R&D Systems). The same plasma sample was used for each evaluation in each case. These values were used to calculate free IL-18 concentrations assuming 1:1 stoichiometry and a dissociation constant of 0.4 nM in a law of mass action equation (33).

### Functional analysis of naive CD4^+^ and CD8^+^ T cells.

For the measurement of senescence-associated β-galactosidase activity, PBMCs were thawed and cultured for 1 hour in RPMI 1640 medium containing 1 mM sodium pyruvate, 1 mM nonessential amino acids, 1 mM L-glutamine, 1% penicillin/streptomycin, 25 mM HEPES, and 0.1 μM bafilomycin (all reagents from Thermo Fisher Scientific). Cells were then incubated for a further 2 hours in the presence of SA-β-Gal Fluorescent Substrate (33 μM, Cell Signaling Technology). For the measurement of proliferation, PBMCs were thawed and stimulated in 96-well plates coated with an anti-CD3 antibody (clone OKT3, BD Pharmingen). After 48 hours, cells were stained with directly conjugated monoclonal antibodies specific for CCR7 (clone 2-L1-A, BD Biosciences), CD4 (clone RPA-T4, BD Biosciences), CD8 (clone RPA-T8, BD Biosciences), and CD45RA (clone HI100, BD Biosciences). Nonviable events were excluded using a LIVE/DEAD Fixable Aqua Dead Cell Stain Kit (Thermo Fisher Scientific). Cells were then fixed/permeabilized using a Foxp3/Transcription Factor Staining Buffer Set (Thermo Fisher Scientific) and stained with a directly conjugated antibody specific for Ki-67 (clone Ki-67, BioLegend). Data were acquired using an LSR Fortessa (BD Biosciences) and analyzed using FlowJo version 10.8.1 (FlowJo LLC).

### In vitro stimulation of antigen-specific CD8^+^ T cells.

Antigen-specific CD8^+^ T cells were stimulated in vitro as described previously (31, 36). PBMCs from healthy HLA-A2^+^ donors were cultured in AIM-V medium (Thermo Fisher Scientific) containing FMS-like tyrosine kinase 3 ligand (FLT3L, 50 ng/mL, R&D Systems). After 24 hours, cells were incubated with the heteroclitic Melan-A/MART-1_26–35_ peptide ELAGIGILTV (ELA, 1 μM), the influenza matrix M1_58–66_ peptide GILGFVFTL (GIL, 0.01 μM), or the SARS-CoV-2 spike protein_269–277_ peptide YLQPRTFLL (YLQ, 0.01 μM) to stimulate de novo (ELA) or recall CD8^+^ T cell responses (GIL or YLQ). Test agents were added at this stage, including patient sera (100% v/v), IL-6 (100 ng/mL, R&D Systems), IL-18 (100 ng/mL, BioLegend), IP-10 (100 ng/mL, R&D Systems), or HGF (100 ng/mL, Miltenyi Biotec). The effects of IL-18 were blocked using IL-18BP (1 μg/mL, BioLegend) and anti–IL-18 receptor (1 μg/mL, IL-18Rɑ/IL1 R5 polyclonal goat IgG, R&D Systems). On day 2, FBS was added at a final v/v ratio of 10%. Medium was replaced every 3 days thereafter with fresh RPMI 1640 medium containing 10% FBS. Antigen-specific CD8^+^ T cells were identified via flow cytometry on day 12 using tetrameric complexes of ELA/HLA-A*02:01, GIL/HLA-A*02:01, or YLQ/HLA-A*02:01 conjugated to BV421 or PE (50). Cells were then fixed/permeabilized using a Transcription Factor Buffer Set (BD Biosciences) and stained with directly conjugated monoclonal antibodies specific for granzyme B (clone GB11, BD Biosciences) or perforin (clone B-D48, BioLegend). Data were acquired using an LSR Fortessa (BD Biosciences) and analyzed using FlowJo version 10.8.1 (FlowJo LLC).

### Statistics.

Simple group comparisons were performed using the Mann-Whitney *U* test or Wilcoxon’s signed-rank test, and correlations were assessed using Spearman’s rank test. All basic statistical analyses were performed using Prism version 9 (GraphPad). The functional profiles of SARS-CoV-2–specific CD4^+^ and CD8^+^ T cells were compared using permutation tests in SPICE version 6 (https://niaid.github.io/spice/).

### Study approval.

The primary study was approved by the Comité de Protection des Personnes at the University Hospital of Bordeaux, France (COLCOV19-BX, NCT04332016). The secondary study was approved by the Institutional Ethics Committee of the National Institutes of Biomedical Innovation, Health and Nutrition (137 and 117-4) and by the Institutional Ethics Committee of The Research Foundation for Microbial Diseases of Osaka University (20-02), Japan. All donors provided written informed consent in accordance with the principles of the Declaration of Helsinki.

### Data availability.

The raw data are available in the Supporting Data Values file and from the corresponding author upon request.

## Author contributions

GA, LP, TN, ABM, DK, KS, YY, FN, DAP, JDM, TY, IP, and VA designed experiments. GA, LP, TN, ABM, DK, MR, YM, and SLL performed experiments. GA, LP, TN, ABM, DK, MR, KS, YM, EW, TY, IP, and VA analyzed data. GA, LP, DAP, TY, IP, and VA wrote the manuscript.

## Supplementary Material

Supplemental data

Supporting data values

## Figures and Tables

**Table 1:**
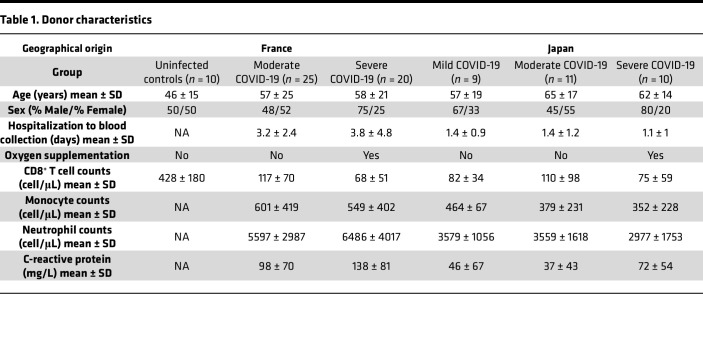
Donor characteristics

## Version 1 01/23/2025

In-Press Preview

## Version 2 02/24/2025

Electronic publication
