# Supplementary material for: Aging and inflammation limit the induction of SARS-CoV-2–specific CD8+ T cell responses in severe COVID-19
Source: JCI Insight. 2025 Jan 23;10(4):e180867. doi: 10.1172/jci.insight.180867 (PMC11949069; doi:10.1172/jci.insight.180867)
Supplement: Supplemental data [file jciinsight-10-180867-s161.pdf]

Ageing and inflammation limit the induction of SARS-CoV-2-specific CD8$^+$ T cell responses in severe COVID-19

Autaa et al.

SUPPLEMENTAL MATERIAL

Supplemental Figure 1.

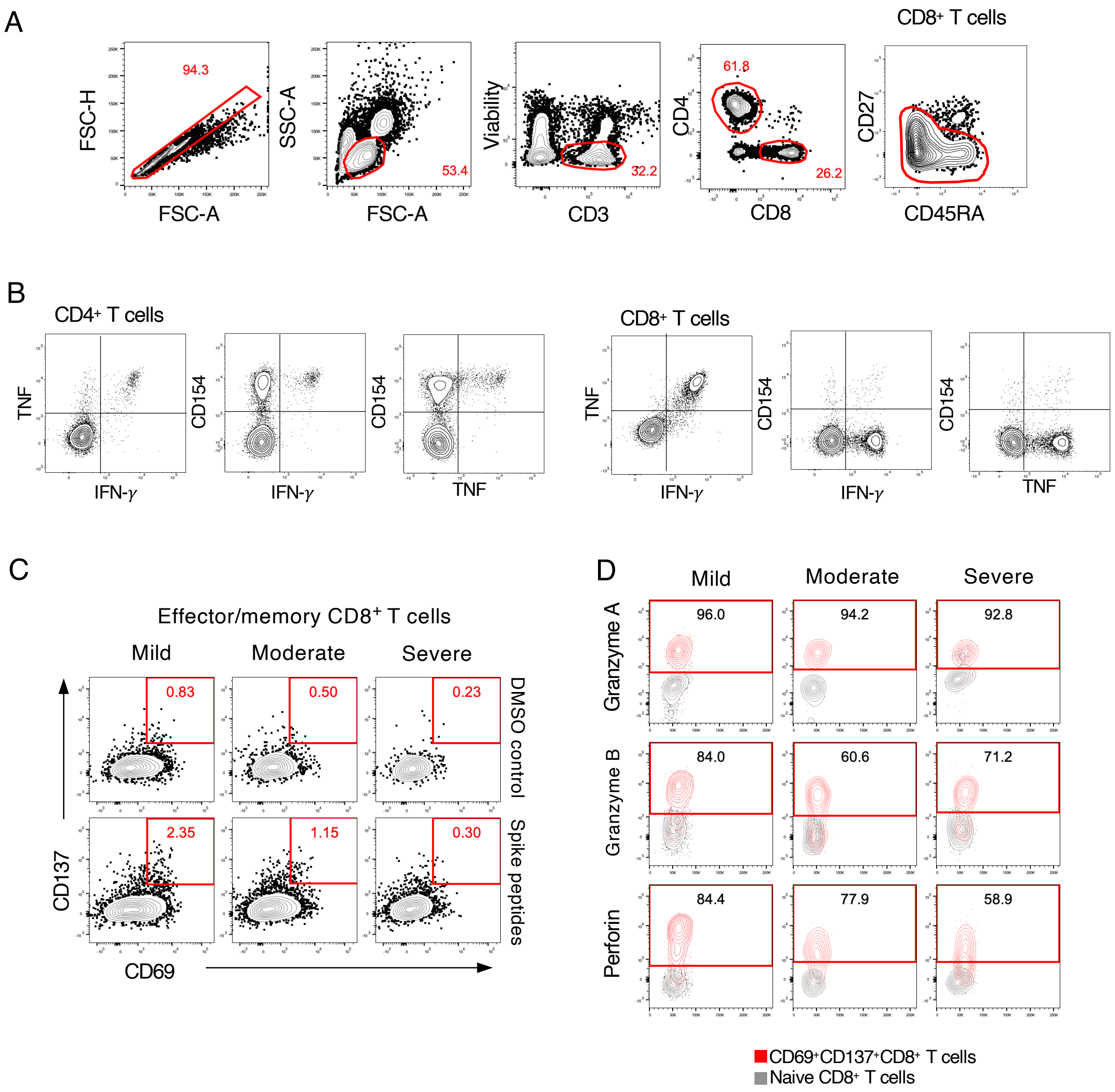

Supplemental Figure 1. Gating strategy for the ex vivo identification of SARS-CoV-2-specific CD4$^+$ and CD8$^+$ T cells in patients with acute COVID-19. (**A**) Representative flow cytometry plots showing the identification of single, viable CD3$^+$ events segregated by lineage

(CD4 versus CD8) and differentiation status (CD27 versus CD45RA). Numbers indicate percentages in the drawn gates. Right: effector/memory cells defined after exclusion of naive events expressing CD27 and CD45RA. (**B**) Representative flow cytometry plots showing the identification of spike-specific $CD4^+$ (left) and $CD8^+$ T cells (right) among patients in the primary cohort via upregulation of the activation marker CD154 and/or intracellular expression of the effector cytokines IFN-γ, TNF, or IL-2. (**C**) Representative flow cytometry plots showing the identification of effector/memory spike-specific $CD8^+$ T cells among patients in the secondary cohort via upregulation of the activation markers CD69 and CD137. Numbers indicate percentages in the drawn gates. (**D**) Representative flow cytometry plots showing the intracellular expression of granzyme A, granzyme B, and perforin among effector/memory $CD8^+$ T cells (red) from patients in the secondary cohort identified via upregulation of the activation markers CD69 and CD137. Comparative analyses are shown for naive $CD8^+$ T cells in each case (gray). Numbers indicate percentages in the drawn gates.

Supplemental Figure 2.

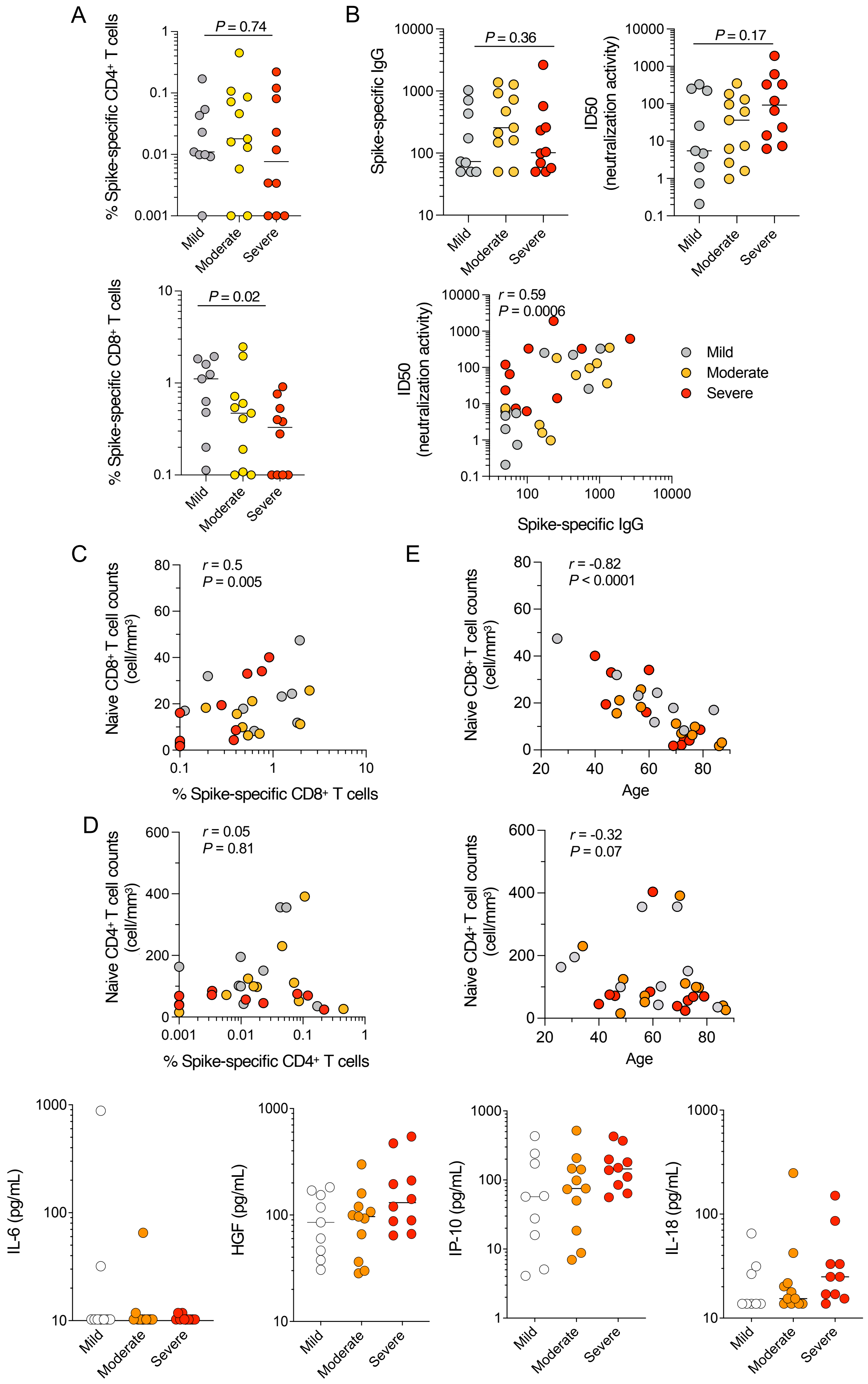

Supplemental Figure 2. Immunological characterization of patients in the secondary cohort with acute COVID-19. (**A**) Frequencies of spike-specific $CD4^+$ and $CD8^+$ T cells ($CD69^+CD137^+$) among patients in the secondary cohort grouped according to disease severity. Each dot represents one donor. Bars indicate median values. Significance was assessed using the Mann–Whitney U test. (**B**) Top: SARS-CoV-2-specific IgG titers (left) and neutralization activity (right) among patients in the secondary cohort grouped according to disease severity. Each dot represents one donor. Bars indicate median values. Significance was assessed using the Mann–Whitney U test. Bottom: correlation between SARS-CoV-2-specific IgG titers and neutralization activity among patients in the secondary cohort with mild, moderate, or severe disease. Each dot represents one donor. Significance was assessed using Spearman's rank test. (**C**) Correlation between the absolute counts of naive $CD8^+$ T cells and the frequencies of spike-specific $CD8^+$ T cells among patients in the secondary cohort with mild, moderate, or severe disease. Each dot represents one donor. Significance was assessed using Spearman's rank test. (**D**) Correlation between the absolute counts of naive $CD4^+$ T cells and the frequencies of spike-specific $CD4^+$ T cells among patients in the secondary cohort with mild, moderate, or severe disease. Each dot represents one donor. Significance was assessed using Spearman's rank test. (**E**) Correlations between the absolute counts of naive $CD8^+$ (top) or $CD4^+$ T cells (bottom) and age among patients in the secondary cohort with mild, moderate, or severe disease. Each dot represents one donor. Significance was assessed using Spearman's rank test. (**F**) Plasma concentrations of IL-6, HGF, IP-10, and IL-18 among patients in the secondary cohort grouped according to disease severity. Each dot represents one donor. Bars indicate median values. Significance was assessed using the Mann–Whitney U test.

Supplemental Figure 3.

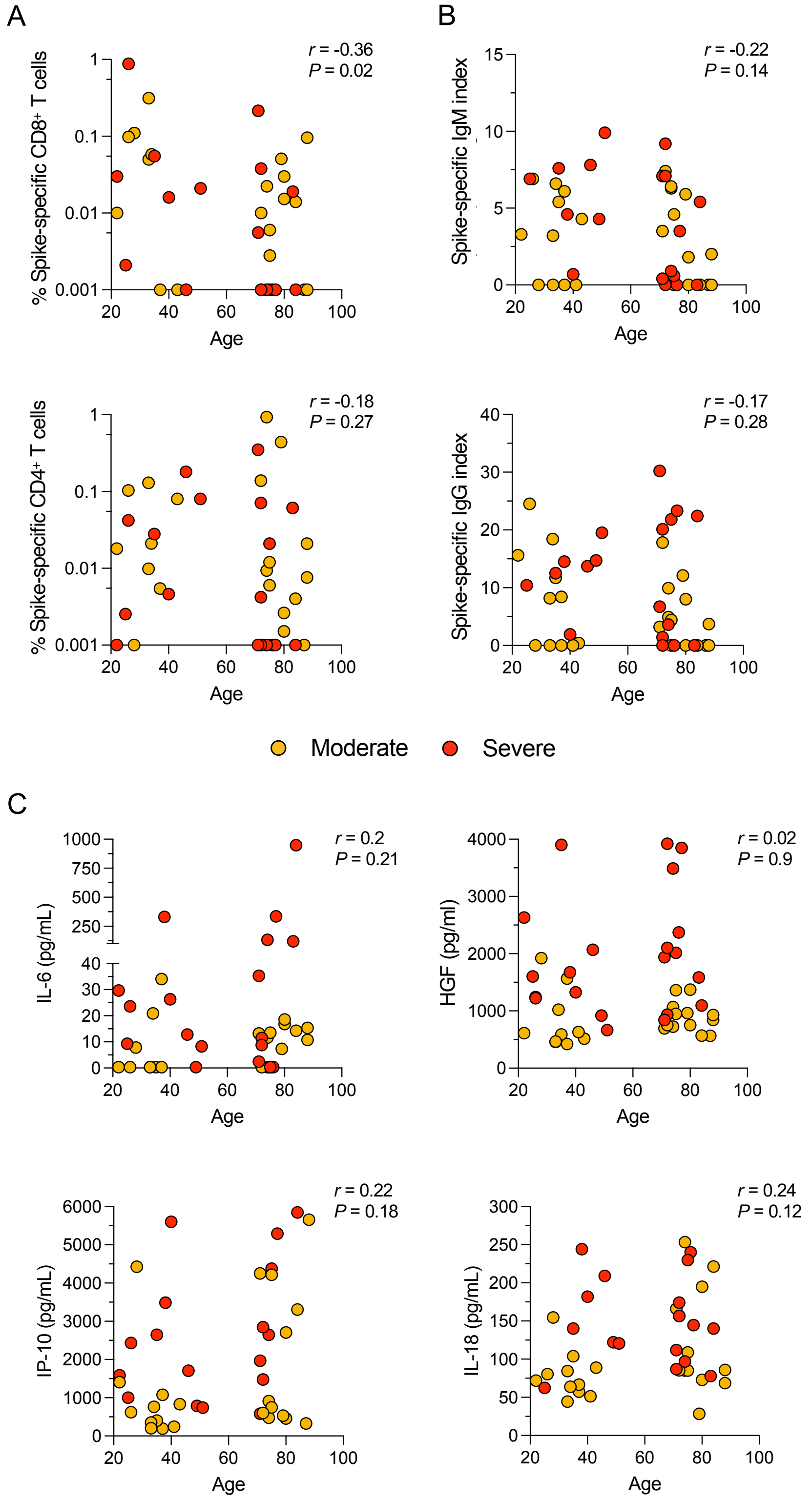

Supplemental Figure 3. Impact of age on immune and inflammatory parameters in patients with acute COVID-19. (A) Correlations between the frequencies of spike-specific $CD8^+$ (top) or $CD4^+$ T cells (bottom) and age among patients in the primary cohort with

moderate or severe disease. Each dot represents one donor. Significance was assessed using Spearman's rank test. (**B**) Correlations between spike-specific IgM (top) or IgG titers (bottom) and age among patients in the primary cohort with moderate or severe disease. Each dot represents one donor. Significance was assessed using Spearman's rank test. (**C**) Correlations between plasma concentrations of IL-6, HGF, IP-10, and IL-18 and age among patients in the primary cohort with moderate or severe disease. Each dot represents one donor. Significance was assessed using Spearman's rank test.

Supplemental Figure 4.

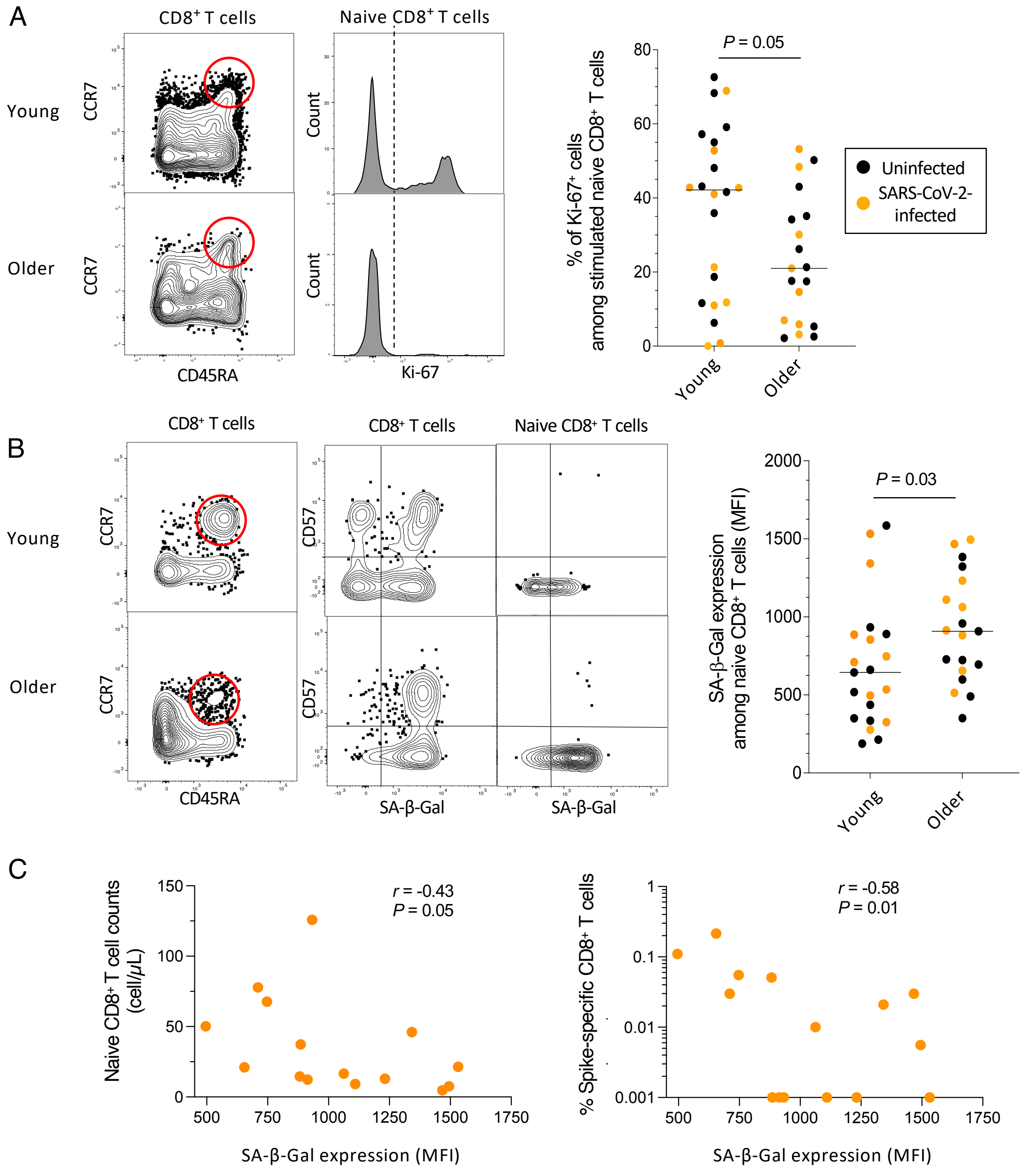

Supplemental Figure 4. Qualitative analysis of naive CD8$^+$ T cells in patients with acute COVID-19. (**A**) Representative flow cytometry plots (left) and summary data (right) showing the expression of Ki-67 among naive CD8$^+$ T cells from young or older individuals with or without acute COVID-19 after stimulation of PBMCs with anti-CD3. Each dot represents one donor. Bars indicate median values. Significance was assessed using the Mann–Whitney U test. (**B**) Representative flow cytometry plots (left) and summary data (right) showing senescence-

associated β-galactosidase (SA-β-Gal) activity among naive $CD8^+$ T cells from young or older individuals with or without acute COVID-19. Each dot represents one donor. Bars indicate median values. Significance was assessed using the Mann–Whitney U test. (**C**) Correlations between SA-β-Gal activity among naive $CD8^+$ T cells and the absolute counts of naive $CD8^+$ T cells (left) or the frequencies of spike-specific $CD8^+$ T cells (right) among patients with acute COVID-19. Each dot represents one donor. Significance was assessed using Spearman's rank test.

Supplemental Figure 5.

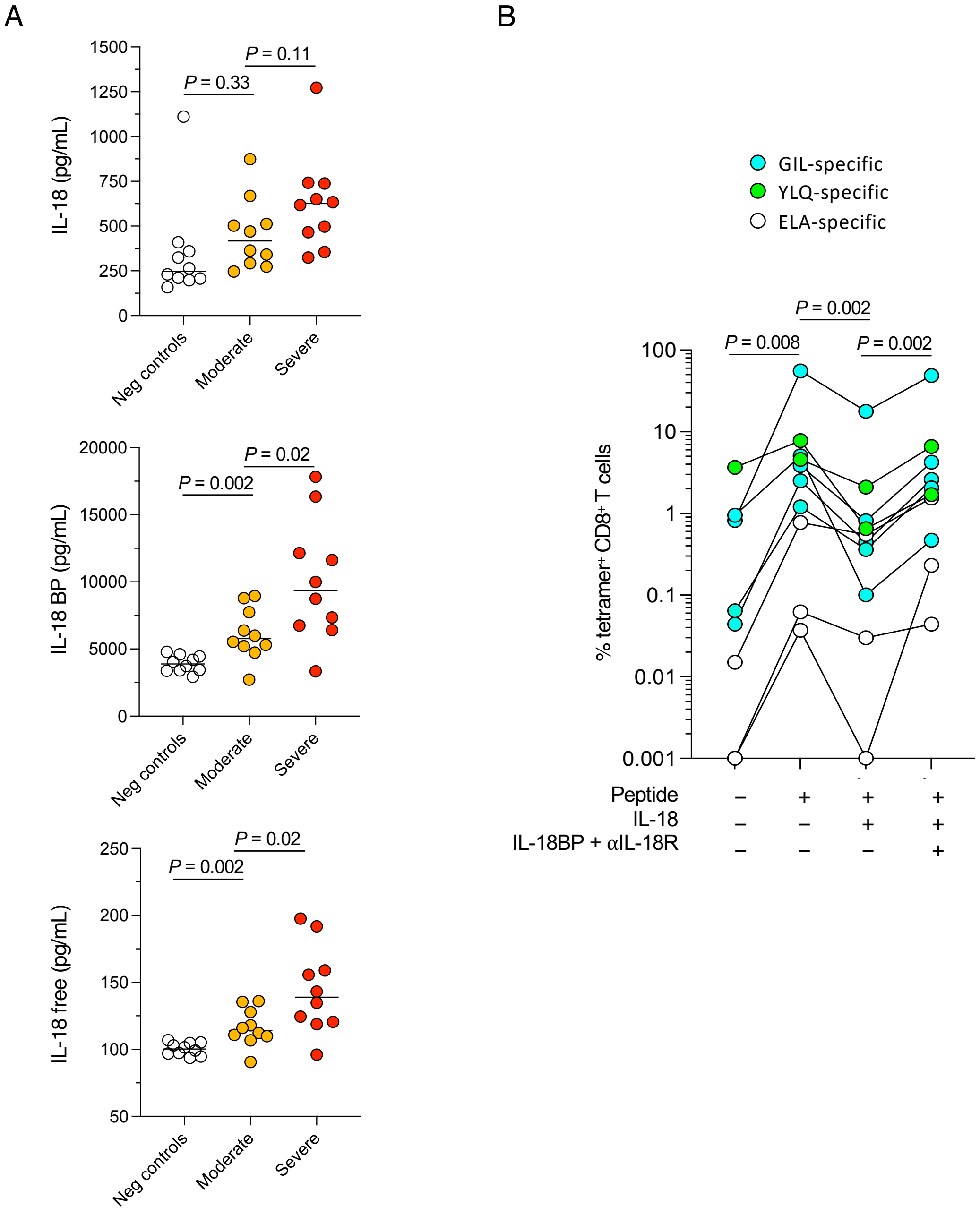

Supplemental Figure 5. Measurement of free IL-18 and the effect of blocking of IL-18 on the expansion of antigen-specific CD8$^+$ T cells in vitro. (**A**) Measured concentrations of total IL-18 and IL-18BP and calculated concentrations of free IL-18 among patients in the primary cohort grouped according to disease severity. Each dot represents one donor. Bars indicate median values. Significance was assessed using the Mann–Whitney U test. (**B**) Frequencies of antigen-specific HLA-A*02:01 tetramer$^+$ CD8$^+$ T cells on day 12 after peptide stimulation of PBMCs from healthy HLA-A2$^+$ donors in absence or presence of IL-18, IL-18BP, and/or an

anti-IL-18 receptor antibody. Each dot represents one donor. Significance was assessed using the Wilcoxon signed rank test.

Supplemental Figure 6.

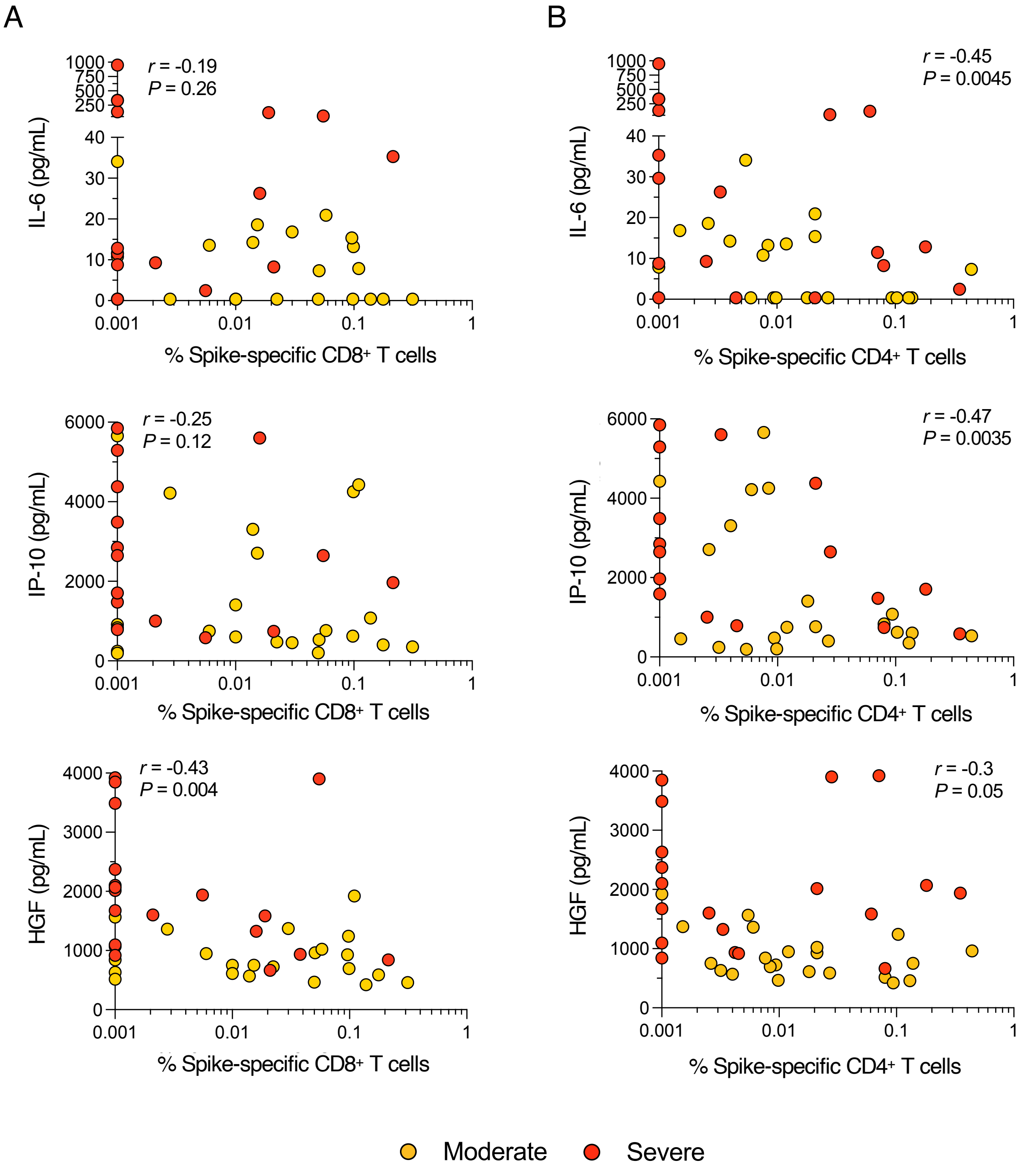

Supplemental Figure 6. Correlations between the frequencies of SARS-CoV-2-specific CD4$^+$ and CD8$^+$ T cells and plasma concentrations of IL-6, IP-10, and HGF. (**A**) Correlations between the frequencies of spike-specific CD8$^+$ T cells and plasma concentrations of IL-6, IP-10, or HGF among patients in the primary cohort with moderate or severe disease. Each dot represents one donor. Significance was assessed using Spearman's rank test. (**B**) Correlations between the frequencies of spike-specific CD4$^+$ T cells and plasma concentrations

of IL-6, IP-10, or HGF among patients in the primary cohort with moderate or severe disease. Each dot represents one donor. Significance was assessed using Spearman's rank test.
